# A case of endoscopic submucosal dissection of esophageal acantholytic Paget’s disease with discontinuously spreading and unclear neoplastic extension

**DOI:** 10.1186/s40792-024-01956-0

**Published:** 2024-06-21

**Authors:** Hidetoshi Satomi, Shingo Ishiguro, Sei Murayama, Takashi Kanesaka, Tomoki Michida, Ryu Ishihara, Keiichiro Honma

**Affiliations:** 1 Department of Diagnostic Pathology and Cytology, Osaka International Cancer Institute, 3-1-69 Otemae, Chuo-Ku, Osaka-City, Osaka 5418567 Japan; 2 PCL Osaka Pathology and Cytology Center, 1-14-17 Nishiawaji, Higashiyodogawa-Ku, Osaka-City, Osaka 5330031 Japan; 3 Department of Gastrointestinal Oncology, Osaka International Cancer Institute, 3-1-69 Otemae, Chuo-Ku, Osaka-City, Osaka 5418567 Japan

**Keywords:** Extramammary Paget's disease, Endoscopic submucosal dissection, Cellular adhesion

## Abstract

**Background:**

Paget's disease (PD) is a carcinoma, in which irregular atypical cells with abundant cytoplasm proliferate mainly within the epithelium and is classified into PD occurring in the breast and extramammary Paget's disease (EMPD) occurring outside the breast. Essentially, extramammary PD is reported as a tumor for which it is difficult for surgeons to properly determine the line of resection.

**Case presentation:**

An 83-year-old male was admitted to our hospital because of roughness of the esophageal epithelium during the follow-up examination for a gastric ulcer. A preoperative biopsy revealed squamous cell carcinoma; therefore, endoscopic submucosal dissection (ESD) was performed.

**Conclusions:**

The characteristic feature in this patient was the distribution of tumor cells and, accordingly, the difficulty in identifying the neoplastic distribution. In this patient, the odd distribution and growth pattern of the tumor cells made it difficult for the operator to identify the distribution of the lesion preoperatively.

## Introduction

Paget's disease (PD) is a carcinoma, in which atypical cells with abundant cytoplasm proliferate mainly within the epithelium. It is classified into PD, in the breast, and extramammary Paget's disease (EMPD) [1]. EMPD is a uncommon tumor that predominantly affects patients in their 50s to 80s, accounting for about 6.5% of PD. There is no consensus on the etiology of EMPD. Its histological diagnosis is challenging, and immunohistochemistry may be needed. The standard treatment is resection [1]. The common sites of EMPD are the vulva and inguinal region [2]. PD of esophagus are relatively rare [2–10].

Rayne et al. reported that some cases of PD were characterized by acantholytic changes in neoplastic cells and described them as anaplastic PD [11]. To date, only two cases of acantholytic anaplastic PD have been reported [5, 9]. Here, we report a case of acantholytic anaplastic PD. In this case, the tumor cells spread discontinuously in the squamous epithelium, forming small tumor foci without distinctive mass formation. Therefore, the distribution of the neoplastic lesion makes it challenging to estimate the localization of the lesion endoscopically. Histologically, the lesion was positive at the surgical margins.

This is a unique case of PD with a unique gross picture and histology, and we believe that knowledge of this kind of PD may provide a clinicopathological evidence for accurate diagnosis and successful endoscopic treatment.

## Case presentation

**Fig. 1:**
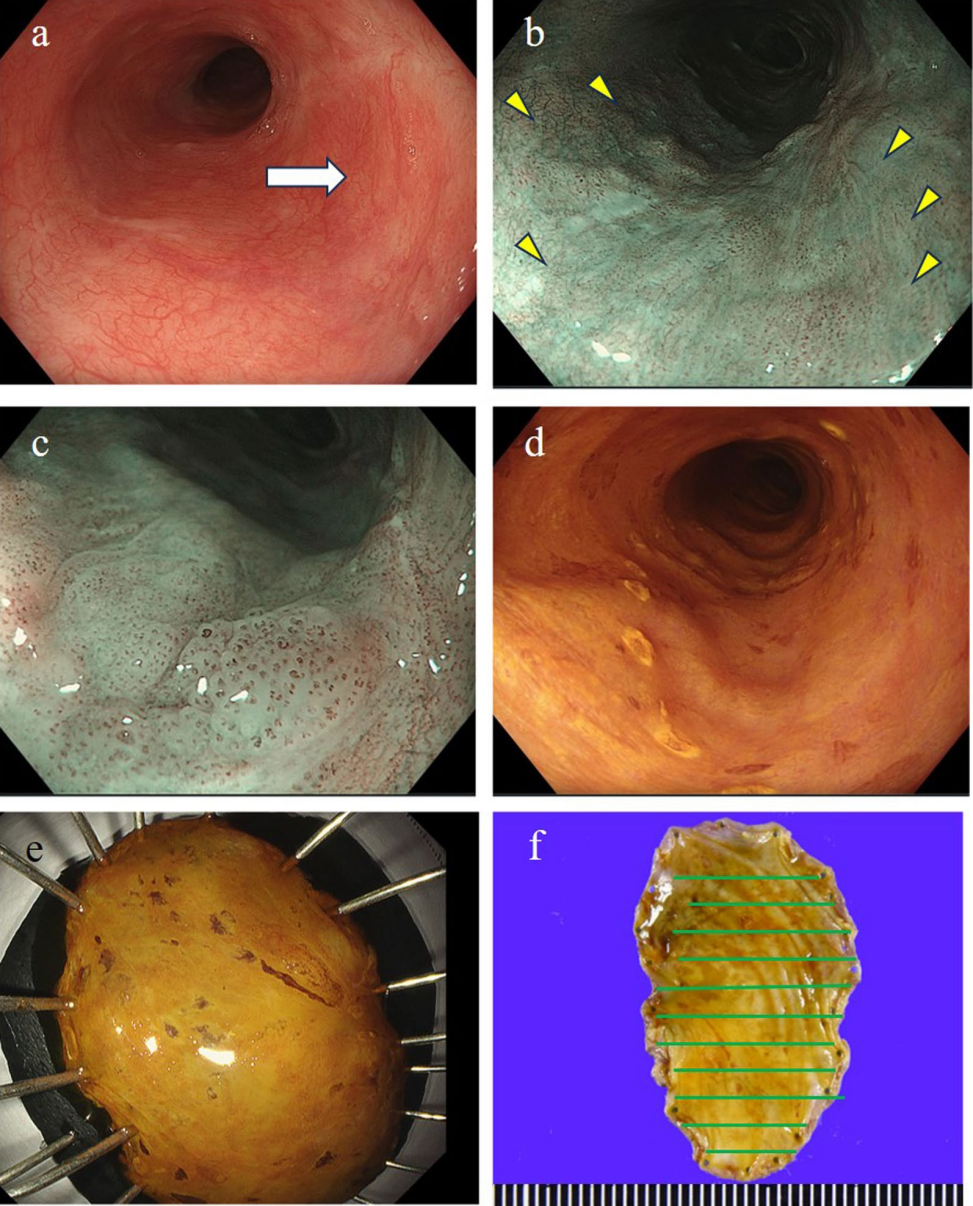
Endoscopic and macroscopic findings of the lesion and specimen. **a** Mucosal irregularities were observed at a site 30 cm from the superior incisal fissure. A low elevated lesion was identified (arrow). **b**, **c** Dotted dilated blood vessels (Type B1) were observed (arrowheads). **d** After marking with iodine staining, however the boundary was unclear and it was difficult to identify the extension of the lesion. **e** The size of the resected specimen is 34 × 18 mm. A low elevated lesion was identified in the middle of the specimen. **f** Iodine staining did not reveal an unstained zone, and it was difficult to determine the extension of the neoplastic lesion grossly. The green ruled line indicates the tumor location

**Fig. 2:**
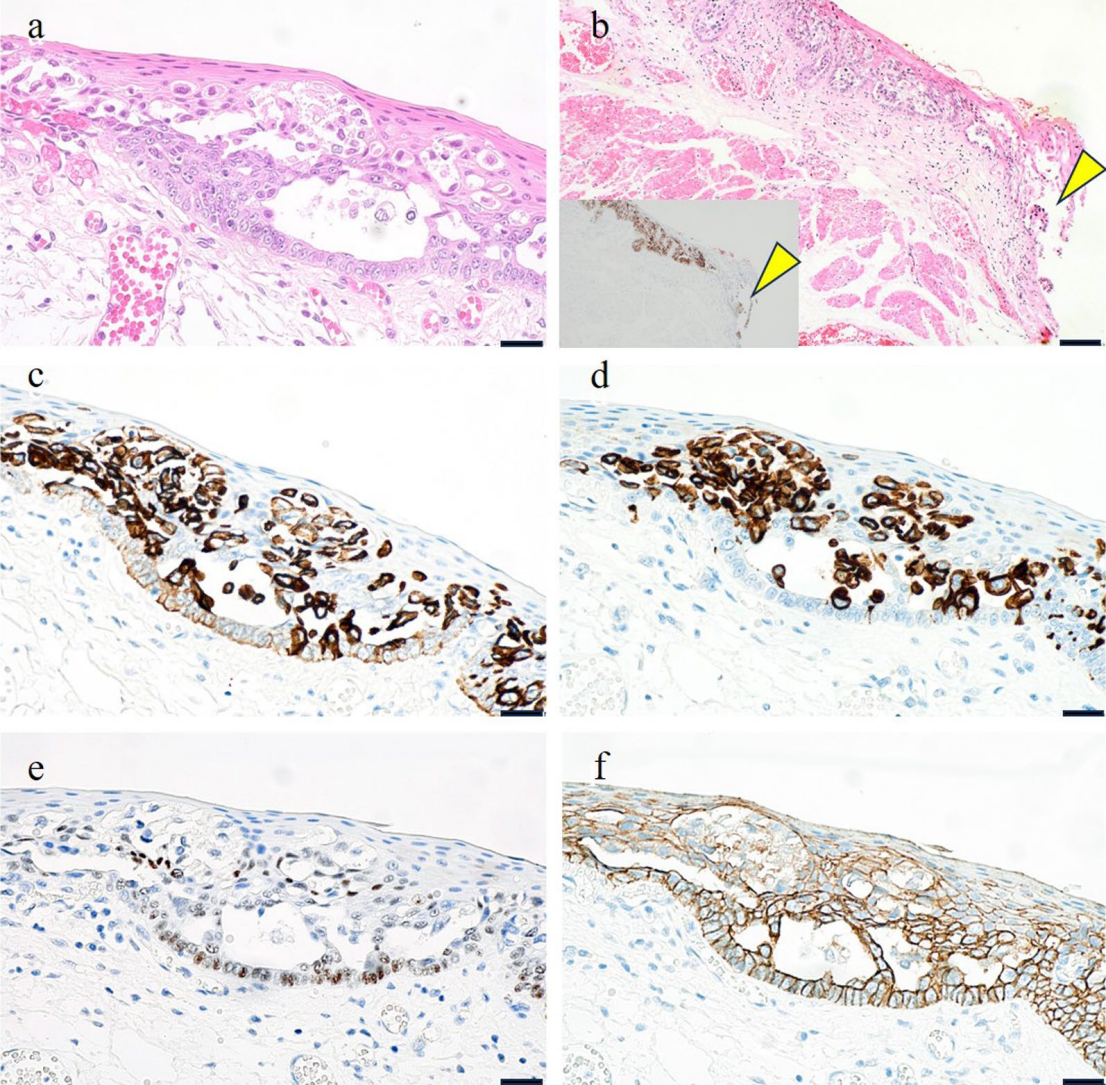
Histological findings of the resected specimen. **a** Hematoxylin and eosin staining of the main lesion of the patient. Tumor cells are seen in the spaces between non-neoplastic squamous epithelium. Cell adhesion of each is loose. **b** Hematoxylin and eosin staining of horizontal margins of the specimen (arrowheads). Tumor cells are exposed on the horizontal surgical margin. Immunohistochemistry shows these findings, too (inset, CAM5.2). **c**–**f** Immunohistochemistry of tumor cells (**c** CAM5.2; **d** CK7; e, p40; **f** E-cadherin). Tumor cells are CAM5.2-positive, CK7-positive and p40-negative. E-cadherin positivity is got weakened (**a**, **c**–**f**, scale bar is 20 μm; **b**, scale bar is 100 μm)

An 83-year-old man. He was admitted to our hospital because of roughness of the esophageal epithelium. The preoperative diagnosis was squamous cell carcinoma due to atypical cells resembling parabasal cells with eosinophilic cytoplasm. However, the specimen was small, and was difficult to confirm the presence of intraepithelial skipped nests as seen in the surgical specimen. Endoscopic submucosal dissection (ESD) was performed. Endoscopic examination revealed a low elevated lesion with flat extension at a site 30 cm from the superior incisal fissure (Fig. 1a). The boundary of the lesion was unclear. Since the lesional border was not well recognized by iodine staining, narrow band imaging (NBI) was used to determine the boundary (Fig. 1b–d). The preoperative diagnosis was cM1, cM2. The resected specimen had a rough appearance (Fig. 1e, f). Histologically, atypical cells had eosinophilic cytoplasm and irregular nuclei. Atypical cells proliferated and spread in the spaces between the discohesive squamous cells (Fig. 2a). Immunohistochemically, the tumor cells were positive for CK7, CAM5.2 (Fig. 2c, d), weakly positive for E-cadherin (Fig. 2f), and negative for p40 (Fig. 2e), p63, and CK20 (results not shown). These findings were consistent with PD (pT1a-EP). Neoplastic cells were exposed on the horizontal surgical margin (Fig. 2b). However, this was a few cells, no additional treatment was added, and 6 months after the surgery, there was no recurrence.

## Discussion

The histochemical findings were consistent with PD, and there were no neoplastic changes in the neighboring organs. Therefore, we diagnosed the patient with primary PD of the esophagus. Esophageal PD is relatively uncommon [2–10]. However, fewer cases could be identified as “true” primary esophageal PD, i.e., those that showed evidence of glandular differentiation and excluded intraepithelial metastases of carcinomas arising in other sites [3–8] (Table 1).

**Table 1:** Characteristics of the primary Paget’s disease of the esophagus with previous reports

Case	References	Age	Sex	Chief complaint	Segment	Localization	Primary treatment	Outcome
1	Haleem et al. [3]	74	F	Dysphagia	Lt	Carcinoma in situ	Esophagogastrectomy	NA
2	Karakök et al. [4]	72	M	Dysphagia	Lt	NA	NA	NA
3	Lin et al. [5]	63	M	Dysphagia	Lt	Lamina propria	Esophagectomy	Recurrence^a^
4	Matsukuma et al. [6]	59	M	NA	U-Mt	Minimal submucosal	NA	NA
5	Mori et al. [7]	53	M	Dysphagia	NA	Intraepithelial	EMR	NA
6	Nonomura et al. [8]	60	M	Dysphagia	NA	Intraepithelial	Esophagogastrectomy	NA
7	This case	83	M	No complaints	Mt	Intraepithelial	ESD	Alive

*Lt* lower thoracic esophagus, *U-Mt* upper to middle thoracic esophagus, *NA* not available, *EMR* endoscopic mucosal resection, *ESD* endoscopic submucosal dissection

^a^Lymphnodal metastatic recurrence

Two cases of extramammary PD with similar findings (one primary esophageal and one primary scrotal) were reported to be acantholytic anaplastic PD [5, 9]. In these two cases, the tumor cells were relatively well defined. However, in the present case, the tumor cells grew sporadically and were isolated within the pre-existing epithelium without forming conspicuous clusters. There have been no previous reports of EMPD with such characteristics.

In our case, immunohistochemical findings suggested E-cadherin depletion in the tumor cells. Therefore, it is possible that the weakening of E-cadherin was the cause of the reduced connectivity between cells and the isolated and scattered distribution of some tumor cells without the formation of large clusters. Deactivation of Cadherin1 has been hypothesized to be the mechanism underlying the loss or weakening of E-cadherin expression. This theory has been proposed for invasive lobular carcinoma and gastric poorly differentiated adenocarcinoma; however, it is not known whether this mechanism is true for PD [12, 13]. Since PD is also an adenocarcinoma, it is assumed to be caused due to similar reason; however, it is desirable to elucidate the mechanism by accumulating cases.

In this case, the distribution of tumor cells also made it difficult to identify the distribution of the lesion preoperatively. PD is reported as a tumor that is difficult for surgeons to determine the line of resection properly because the boundaries of the lesion are not clear, and the tumor tends to spread unexpectedly beyond macroscopic tumor boundaries [14]. Iodine staining in esophageal endoscopy utilizes the chemical reaction of iodine and glycogen in the squamous cells of the esophageal epithelium. Iodine staining can identify cancer in only a little more than 80% of cases [15]. However, thinning or loss of glycogen-containing non-neoplastic esophageal squamous epithelium may result in reduced iodine staining and may be unstained. To identify the neoplastic lesion, the boundary between the non-neoplastic squamous epithelium and tumor cells must be clear. However, the scattered distribution of tumor cells, as in this case, may have contributed to the difficulty of localization diagnosis. The prognosis for EMPD depends on the surgical margins [1]. This may also be true for primary esophageal PD. Although it is difficult to identify the extent of PD in primary esophageal lesions by iodine staining, this case demonstrated that the neoplastic boundaries can be almost well identified by the vascular changes observed by NBI. The standard treatment of esophageal PD is a surgical procedure similar to that for conventional squamous cell carcinoma [3, 5, 8]. There are high expectations that endoscopic treatment can complete the treatment for PD.

## Conclusions

We report a case in which the differentiation tendency, distribution of tumor cells made it difficult to determine the extent of the lesion, and thus, made curative treatment difficult. In cases the location of the lesion is difficult to determine endoscopically, it is important to consider the possibility of esophageal lesions forming indistinct boundaries, especially PD.

## Data Availability

The availability of the data used in this case is subject to confirmation by the journal or the authors. For more information on data availability and access procedures, please contact the journal or corresponding author.

## Abbreviations

PD: Paget’s disease
EMPD: Extramammary Paget’s disease
ESD: Endoscopic submucosal dissection
NBI: Narrow band imaging
CK: Cytokeratin
